# Effects of lidocaine incorporation (without epinephrine) on pain and 2-week complications of botulinum toxin: a double-blind randomized placebo-controlled clinical trial

**DOI:** 10.1038/s41598-023-34973-4

**Published:** 2023-05-14

**Authors:** Farzin Sarkarat, Diba Bagheri, Roozbeh Kahali, Ali Fateh, Vahid Rakhshan

**Affiliations:** 1 Department of Oral and Maxillofacial surgery, Gulf Medical University, Ajman, UAE; 2 Department of Oral and Maxillofacial Surgery, Islamic Azad University of Medical Sciences, Tehran, Iran; 3 Unaffiliated, Tehran, Iran; 4 Department of Cognitive Neuroscience, Institute for Cognitive Science Studies, Tehran, Iran

**Keywords:** Randomized controlled trials, Adverse effects, Pain management, Placebo effect, Medical research, Drug development

## Abstract

No study has assessed the effects of the incorporation of isolated lidocaine into botulinum toxin for reducing its pain or complications. Studies on the dilution of botulinum toxin with other materials are as well extremely few, small, and limited methodologically. Therefore, we aimed to evaluate, for the first time, the effects of the incorporation of lidocaine alone into botulinum toxin type A on post-injection pain and complications. In this 2-week prospective, multicenter, double-blind randomized placebo-controlled clinical trial, 729 participants (667 females) were enrolled. They were randomized into placebo and lidocaine dilutions (about 2:1), and then into two brands of toxins (Dysport versus Xeomin). Hence, there were 4 subgroups. In the 2 experimental subgroups, botulinum toxin was diluted with 2% lidocaine without adrenaline; in the 2 control subgroups, botulinum toxin was diluted with normal saline as a placebo. After injection, the pain level was recorded (as an 11-scale numerical rating scale from 0 to 10). After 2 weeks, post-injection complications were assessed based on the participants’ reports and the surgeon’s observations. Data were analyzed using 3-way ANCOVA, multiple binary logistic regression, and bivariable analyses (α = 0.05, β ≤ 0.1). The mean ± SD pain levels in the lidocaine group (n = 263) and the placebo group (n = 466) were 3.51 ± 2.04 and 4.15 ± 2.35, respectively. The mean ± SD pain levels in the subgroups ‘Xeomin-Lidocaine (n = 61), Dysport-Lidocaine (n = 202), Xeomin-Placebo (n = 133), and Dysport-Placebo (n = 333)’ were respectively 3.39 ± 1.86, 3.55 ± 2.09, 4.61 ± 2.49, and 3.97 ± 2.24. Lidocaine incorporation (*P* = 0.001), Dysport brand (*P* = 0.030), and younger age (*P* = 0.032) [but not sex (*P* = 0.406)] reduced pain. The only significant findings for 2-week complications were for the associations observed between aging with increased asymmetry (*P* = 0.022, OR = 1.032) and a need for a retouch (*P* = 0.039, OR = 1.021). Botulinum toxin dilution with lidocaine alone (without adrenaline or other ingredients) can reduce pain without affecting postinjection complications. Toxin brands may cause different extents of pain. Aging, but not sex, may increase pain. Two-week complications were not affected by any factors, except aging in the case of asymmetry and the need for a botulinum toxin retouch.

## Introduction

After the discovery of the blocking effect of botulinum toxin on acetylcholine release and neuromuscular transmission^1^, it has been used for diverse purposes including treatments for strabismus, facial asymmetry caused by paralysis of the facial nerve, blepharospasm, cervical dystonia, migraines, and even hyperhidrosis and overactive bladder^2–11^. Botulinum toxin type A (BTX-A) has become quite prevalent in the field of cosmetics because it has a low risk of complications and it is minimally invasive^3,12,13^. BTX-A is among the most common cosmetic routines for the treatment of forehead and lateral periorbital rhytides as well as glabellar frown lines^3,14^.

However, the use of botulinum toxin is not without problem^15^. The pain of injection is the most common complication reported by patients, which may deter some patients^15^. Additionally, sometimes the toxin can spread around and unintentionally paralyze unwanted muscles, resulting in complications such as ptosis^3,16^. Another challenge is the high vascularization of the areas commonly injected (the forehead, glabella region, and eyes); this increases the risk of toxin washout at the targeted muscles and inadvertent spread of the toxin into neighboring muscles^3,17–20^. To address these problems, some authors have suggested the incorporation of commonly available lidocaine hydrochloride with epinephrine into the botulinum toxin^3,18^. This way, lidocaine may reduce the pain of the toxin, while the vasoconstrictor adrenalin may reduce the spread of the toxin into unwanted areas while at the same time reducing the washout of the toxin and lidocaine.

Lidocaine, being a vasodilator, might have extra clinical advantages beyond pain reduction as well. Some surgeons and dermatologists suggest that the incorporation of lidocaine might enhance the efficacy or longevity of the botulinum toxin effect^3^. Some clinicians suggest that excluding the vasoconstrictor adrenalin may increase this desirable effect of lidocaine even more. However, if the vasoconstrictor epinephrine increases the predictability, perhaps the vasodilator lidocaine without epinephrine^21^ might reduce the predictability and make the botulinum toxin spread around the injection area more than it should. It raises concerns regarding iatrogenic complications without epinephrin. Also, it is possible that without adrenaline, lidocaine is rapidly washed out of the area and becomes attenuated below the effective concentration, losing its anesthetic effects. This is clinically important and warrants assessment.

Despite the abovementioned points, no studies have assessed the effect of the incorporation of isolated lidocaine into botulinum toxin on the intensity of botulinum pain or complications. Due to the importance of this subject, and in light of the abovementioned shortcomings in the literature, and since there was no study on the incorporation of lidocaine without any additives such as epinephrine or sodium bicarbonate, we tested whether the addition of isolated lidocaine can reduce the pain of BTX-A. Also, we investigated whether or not the addition of lidocaine without adrenaline may cause more complications. For this purpose, a very large-scale randomized clinical trial was conducted. The null hypotheses were the lack of any effect of lidocaine and the brands of botulinum A toxin (as well as participants’ sex and age) on the injection pain or 2-week post-injection complications.

## Materials and methods

This study was a prospective, parallel-arm, 4-arm, multicenter, triple-blind randomized, placebo-controlled clinical trial performed from 2020 to 2022 on 729 participants in a private clinic and a hospital in Tehran, Iran with a treatment/placebo allocation ratio of about 1:2. All participants were briefed completely before the study and signed written informed consent. The ethics of this study was approved by two independent bodies: the research committee of the Islamic Azad University (ethics code: IR.IAU.DENTAL.REC.1399.301, date: 07/03/2021) as well as an international organization responsible for examining, approving, and registering randomized clinical trials (IRCT.ir) following the Helsinki declaration (RCT code: IRCT20210401050804N1, date: 26/04/2021). After the registration of the study, the sample size was augmented to 729 from an original size of 96 individuals, per more conservative criteria to ensure high power, reliability, and generalizability (given that the treatment was harmless; detailed below). The experiments began on 25/04/2021 and continued until May 2022.

### Eligibility criteria and the sample

Included were all patients who had indications for BTX-A injection in the forehead area and had been referred to BouAli Hospital, Tehran, and a private clinic at the time of the examination and who did not have a previous history of an unpleasant postinjection issue. Each person needed to have symmetric mimetic forehead muscle function. Patients with any history of allergy to botulinum toxin type A or lidocaine as well as any history of a neuromuscular disorder would be excluded. Initially, after filling out the file for patients and taking a medical history, all patients with a previous unpleasant history (including allergies) and all patients with asymmetry in injection sites, eyelid ptosis, and diplopia were excluded.

Diagnosis of diplopia was made according to the patient’s statements; asymmetry and ptosis were diagnosed via observation by a specialist physician. Only patients with full data were included; in other words, if a patient would not attend the follow-up session 2 weeks after the injection, the recorded pain score would be discarded as well. The patients lost to follow-up would be replaced by new patients in order to reach the desired sample size.

### Interventions

Based on the dilution materials and the Botulinum toxin brands, there were 4 subgroups:

*A*: Each participant received 100 units of BTX-A (Dysport, Ipsen, France) at 20 injection points, combined with 1 ml of normal saline without preservatives.

*B*: Each person received 100 units of BTX-A (Dysport) combined with 1 ml of lidocaine 2% without epinephrine at 20 injection points.

*C*: Each individual received 50 units of BTX-A (Xeomin, Merz, Germany) combined with 1 ml of normal saline without preservatives at 20 injection points.

*D*: Each patient received 50 units of BTX-A (Xeomin) attenuated with 1 ml of lidocaine 2% without epinephrine.

The allocation ratio of experimental-to-placebo groups was about 1:2. The allocation ratio of the botulinum toxin brands was determined according to their market availability, i.e. subgroups utilizing the Dysport brand were larger than Xeomin subgroups because Dysport was more available than Xeomin. The amount of botulinum toxin to be used for each patient was instructed by the manufacturer.

#### Injection protocol

**Figure 1:**
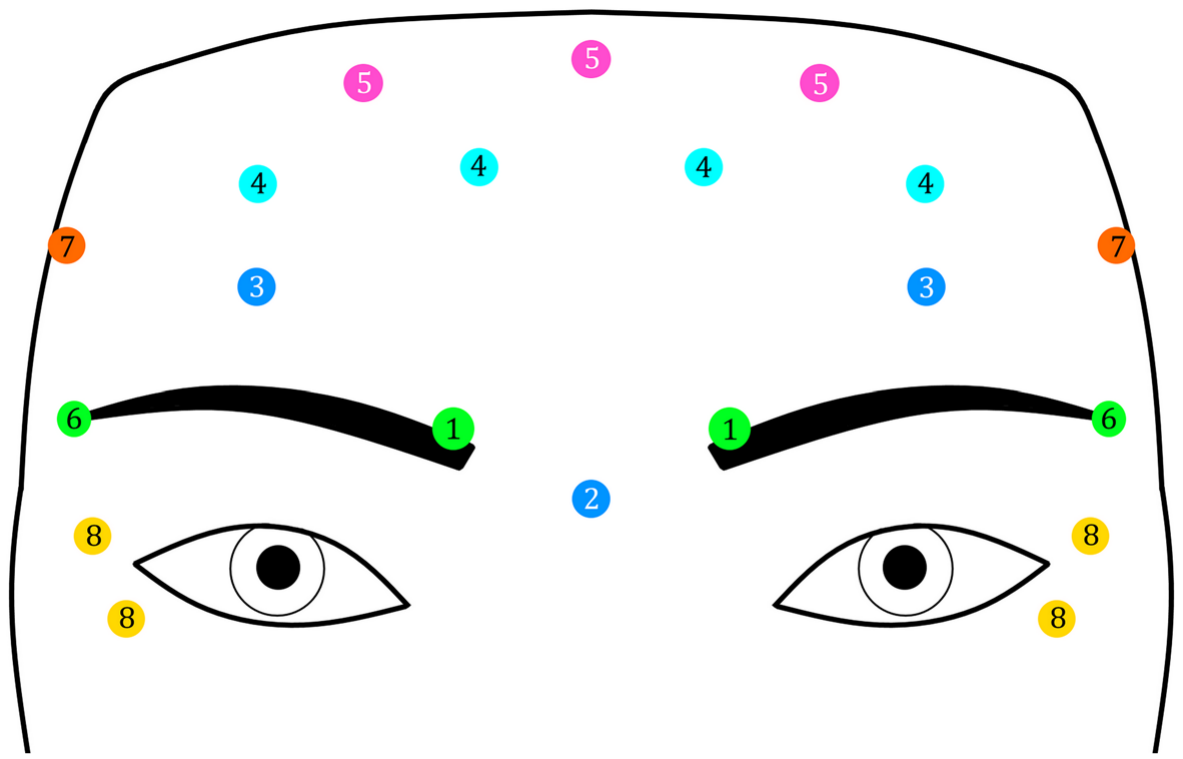
Injection sites: 1. Edge of the corrugator muscle; 2. The distance between the previous 2 points on the nose; 3. About 1 cm above the supraorbital notch; 4. About 1 cm above point #3 on each side, two new points were selected on the frontal muscle with equal distances; 5. About 1 cm above points #4 and between these points, three new points with equal distances were selected on the frontal muscle; 6. The tail of the eyebrow; 7. The edge of the temporal line; 8. Around the eye at the location of the orbicularis oculi muscle on the lateral rim of the eye, two points were selected.

The dilution of botulinum toxin was performed by an experienced technician. Injections were performed in an upright position by an experienced surgeon. This was considered safe because for the dilution with normal saline, we followed the exact instructions of the manufacturers (available in product packages), and for the dilution with lidocaine, previous numerous studies on lidocaine in combination with different materials or alone have found it safe^22^. A 29-gauge needle was used in all subgroups (Terumo K-Pack®II, Japan). In the placebo group, in which botulinum toxin was diluted with normal saline, the normal saline was a 9% injectable sodium chloride solution without any preservatives (Injectable and Pharmaceutical Products, Tehran, Iran). In the experimental group, in which botulinum toxin was diluted with lidocaine, the lidocaine in use was 2% with a preservative (Pasteur Institute, Tehran, Iran). The injection was performed carefully by an experienced surgeon for each person at 20 points (for Dysport: 5 units each, 100 units in total; for Xeomin: 2.5 units each, 50 units in total, Fig. 1). These were standard points recommended in textbooks, and hence effective and safe^23^. The amount of injected toxin from each brand was in accordance with its manufacturer’s instructions.

### Sample size

Since there was no comparative study on the pain intensity after the injection of BTX-A at the time of designing this research, no effect size was available from the literature. Therefore, the authors assumed a conservative effect size (0.25) as well as a high power (90%), resulting in a sample size of 676 participants. The sample size was augmented to 730 patients to offset additional variables besides pain (i.e. 2-week complications). Since there was no limit on the time frame of the study and also since there was a large number of individuals agreeing to participate, we did not anticipate any particular issues in curating the needed data.

### Randomization and blinding

The study was triple-blind, meaning that the surgeon, the participant, and the observer were unaware of the materials and grouping: The surgeon administering the botulinum toxin was blinded to the experimental/placebo assignments and the botulinum brands. Although the numbers of units used from the brands differed, their volumes after dilution would be similar and undetectable from each other. The observers were blinded to the treatment/control assignments and to the botulinum brands as well. The patients were blinded to the grouping as well.

All documents and questionnaires were coded. The coded documents were kept in sealed envelopes. The person who did the randomization also performed the concealment. She was not involved in any of the examinations.

The participants were randomized using a computer program at two levels (Excel, Microsoft, Redmond, Washington, USA). First, they were randomized into one of the two groups (experimental versus placebo) with a ratio of about 1:2. Since the study was large and multicentered, and since many people could be excluded or lost to follow-up, an exact ratio of 1:2 was not necessitated. After the first randomization, the individuals were randomized into either of the two brands of BTX-A with a ratio similar to the market share and availability of these brands. The syringes were coded and given to the surgeon who injected the botulinum toxin. The surgeon, who was the same person in all groups, performed the injection and completed the form without knowing the botulinum toxin brand and the dilution material.

### Outcomes

#### Primary outcome

The post-injection pain was assessed using an 11-level numerical rating scale questionnaire (0 representing no pain and 10 representing maximum and intolerable pain needing emergency attention).

#### Secondary outcomes

Two weeks after injection, ptosis (assessed by observation), asymmetry (through observation), diplopia (assessed by asking the participant), and any need for a botulinum toxin retouch (expressed by the patient) were recorded. Ptosis of the eyelid is one of the most common ocular complications associated with botulinum toxin injection that appears in the first 2 weeks after botulinum injection^24^. Ptosis refers to drooping of the upper eyelid and can affect one or both eyelids. In the mild form, drooping eyelids are above the pupil of the eye, but in more severe cases drooping eyelids cover a part of the pupil so that it reduces the upper visual amplitude^24^. Diplopia is another ocular complication associated with botulinum toxin injection. Diplopia is secondary to extraocular muscle dysfunction. The patient complains of diplopia resulting from the injection, usually in the follow-up 1 week after the injection, which occurs following the paralysis of the lateral rectus muscle^24^. Eyebrow asymmetry is sometimes seen in the upper face third at the tail of the eyebrows, following the injection into the frontalis muscle to treat hyperkinetic forehead lines^24^.

### Statistical analysis

Descriptive statistics and 95% confidence intervals (CIs) were calculated for the pain levels in different groups and subgroups. An independent-samples t-test was used to compare the main groups with each other in terms of their pain and age. Furthermore, a 3-way analysis of covariance (ANCOVA) was used to assess the simultaneous effects of the independent variables (lidocaine incorporation, the brand of botulinum toxin, and participants’ age and sex) on postinjection pain. Age was a continuous variable recorded as an integer number; it was without any grouping and with a unit of change of 1 year. A Pearson correlation coefficient and a partial correlation coefficient were used to examine the correlation between patients’ age (continuous) and their pain levels. Since the correlations were significant, descriptive statistics and 95% CIs were calculated for patients’ pain in different decades of life (as discrete age groups). As well, a histogram was drawn to show the distribution of patients’ ages. For the assessment of the effects of the same 4 independent variables on each of the 2-week post-injection complications, a point-biserial correlation coefficient, a Fisher, and a multiple binary logistic regression were used. Again, the variable “age” was an integer continuous variable without grouping. However, since its role became significant in some complications, the incidences of complications in different life decades (as discrete groups) were summarized as well. The level of significance was set at 0.05.

### Ethics approval and consent to participate

The participants were briefed completely before the study and signed written informed consents. The ethics of this study was approved by two independent bodies: the research committee of the Islamic Azad University (ethics code: IR.IAU.DENTAL.REC.1399.301, date: 07/03/2021) as well as an international organization responsible for examining, approving, and registering randomized clinical trials (IRCT.ir) in accordance with the Helsinki declaration (RCT code: IRCT20210401050804N1, date: 26/04/2021).

## Results

**Figure 2:**
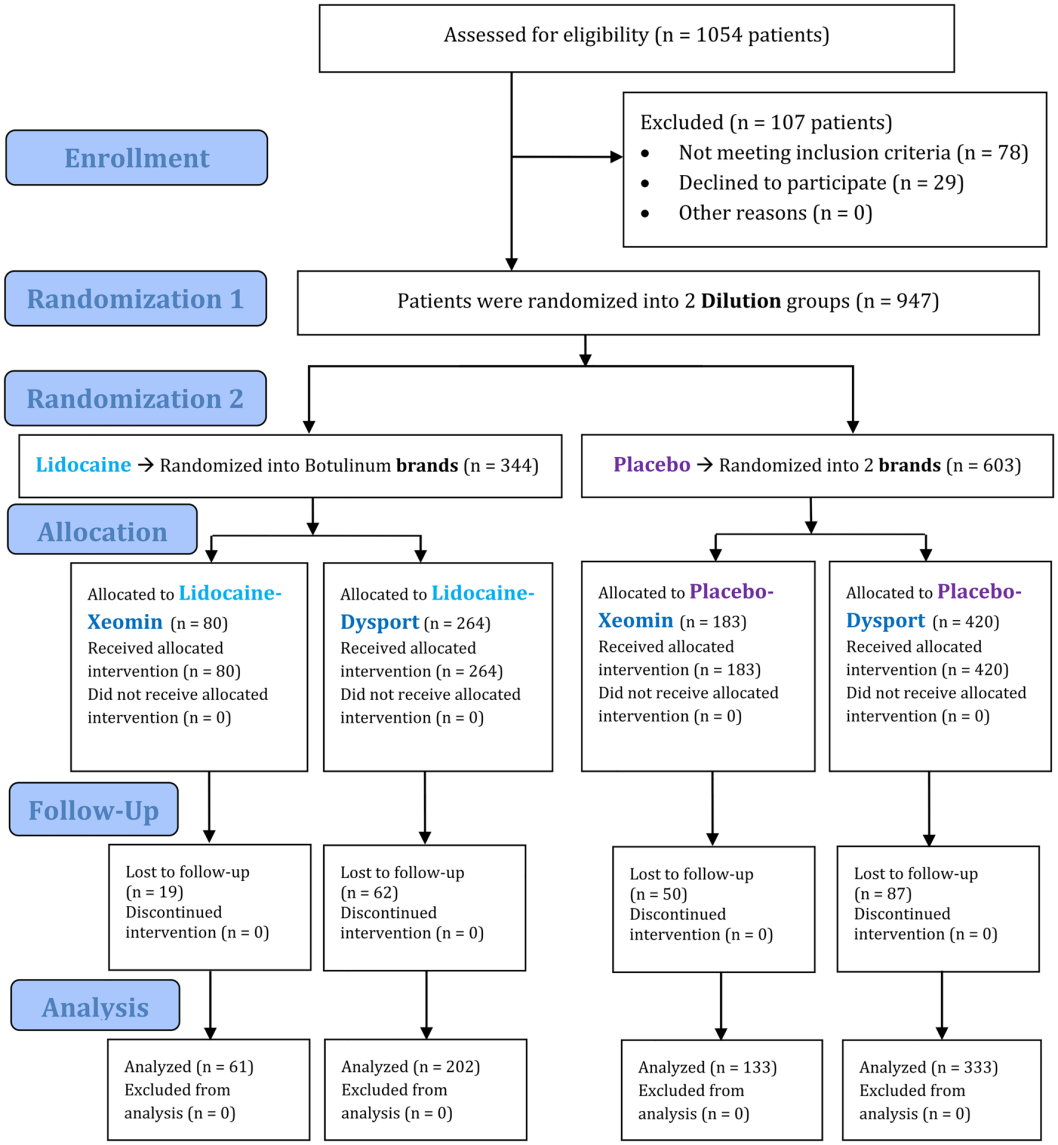
Participants’ flow diagram.

After examining and following up 1054 participants, 729 were approved and enrolled in the study (Fig. 2). There were 667 females and 62 males. A total of 535 Dysport botulinum toxins were used (43 in males, 492 in females). The number of Xeomin toxins in use was 194 (19 in males, 175 in females). Of the 492 Dysport toxins used in females, 306 were diluted with saline while 186 were diluted with lidocaine. Of the 175 Xeomin toxins used in females, 119 were diluted with saline while 56 were diluted with lidocaine. Of the 43 Dysport toxins used in males, 27 were diluted with saline while 16 were diluted with lidocaine. Of the 19 Xeomin toxins used in males, 14 were diluted with saline while 5 were diluted with lidocaine. The participants’ mean age was 42.20 ± 10.79. The mean ages of 62 males and 667 females were respectively 43.10 ± 11.77 years (range: 25 – 70) and 42.11 ± 10.70 years (range: 21–81); the t-test did not detect a significant difference between the mean ages of males and females (*P* = 0.492). The mean ages of participants in different groups are presented in Table 1; the mean ages as well did not show significant differences between the sexes in each of the 4 subgroups (Table 1). Both lidocaine and placebo groups were balanced in terms of sex (chi-square, *P* = 0.703). No harm was identified in this study outside the rare complications of botulinum toxin injection. The trial ended after reaching the desired sample size.

**Table 1:** Descriptive statistics and 95% CIs for participants’ ages.

Brand	Dilution	Sex	N	Mean	SD	95% CI		Min	Max	*P*
Xeomin	Lidocaine	Female	56	37.39	6.76	35.58	39.20	27	55	0.392
		Male	5	40.20	9.42	28.51	51.89	28	53	
		Total	61	37.62	6.96	35.84	39.41	27	55	
	Saline (placebo)	Female	119	43.48	11.61	41.37	45.59	27	81	0.068
		Male	14	37.50	10.34	31.53	43.47	25	56	
		Total	133	42.85	11.59	40.86	44.84	25	81	
Dysport	Lidocaine	Female	186	42.19	9.86	40.76	43.61	25	77	0.700
		Male	16	43.19	10.86	37.40	48.97	25	64	
		Total	202	42.27	9.91	40.89	43.64	25	77	
	Saline (placebo)	Female	306	42.40	11.21	41.14	43.66	21	75	0.073
		Male	27	46.48	12.62	41.49	51.47	26	70	
		Total	333	42.73	11.36	41.50	43.95	21	75	

The *P* values are calculated using the *t* test by comparing the ages of men versus women within each of the 4 subgroups.

*SD* standard deviation, *CI* confidence interval, *Min* minimum, *Max* maximum.

### Pain

**Figure 3:**
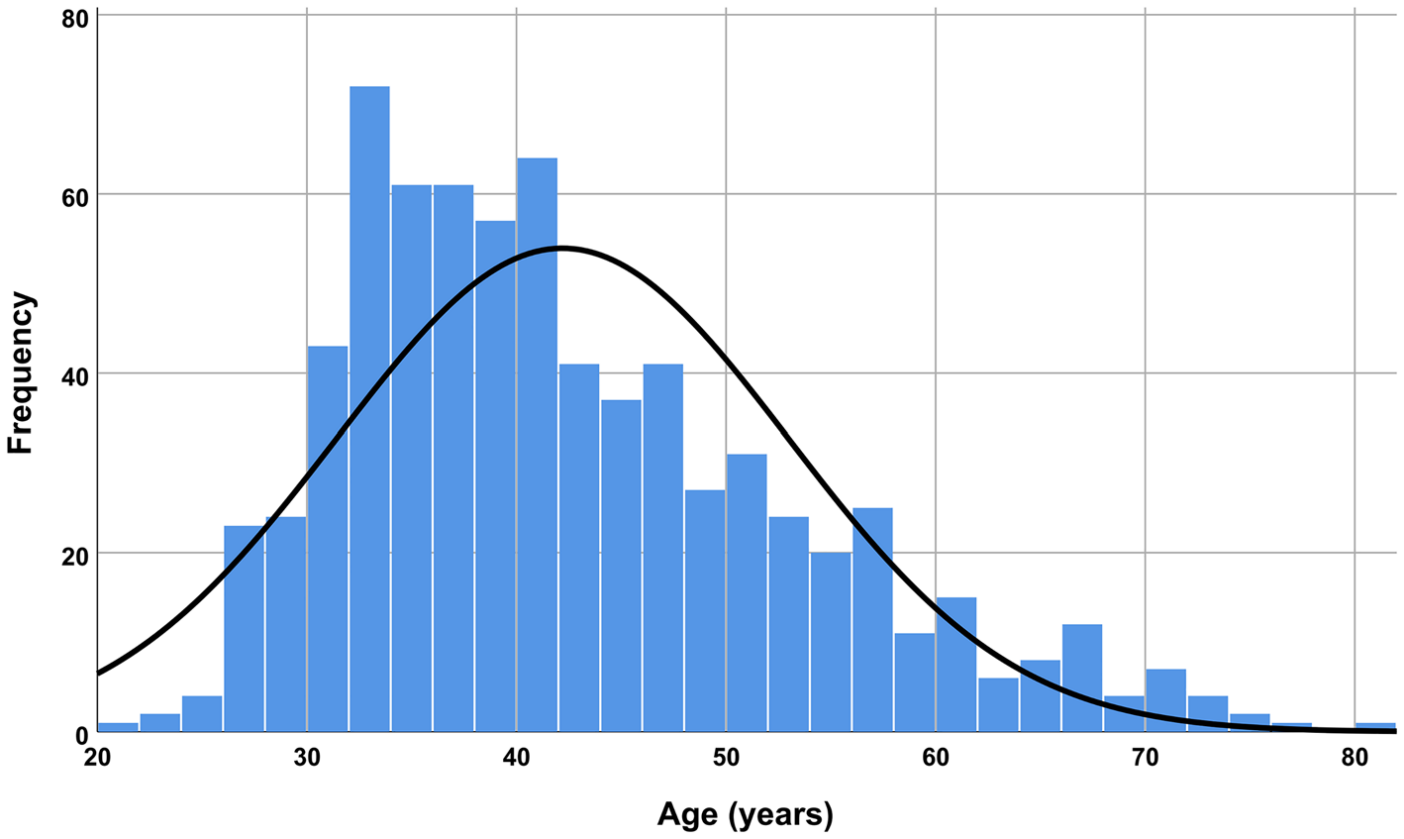
A histogram of the included participants’ ages.

The overall pain level was 3.92 ± 2.246 (range: 0–10, 95% CI 3.76–4.09, Fig. 3, Tables 2 and 3). The *t* test showed significant reductions in pain levels observed in the lidocaine group (both toxin brands combined) compared to the placebo (both brands combined) and pain reductions in the Dysport group compared with Xeomin. However, no significant difference was observed between the sexes (Table 2).

**Table 2:** Descriptive statistics and 95% CIs for pain levels in the main groups, as well as the results of the *t* test comparing the main groups with each other.

Variable	Levels	N	Mean	SD	95% CI		Min	Max	*P*
Dilution	Lidocaine	263	3.51	2.04	3.27	3.76	0	9	**0.0002**
	Saline (placebo)	466	4.15	2.35	3.94	4.36	1	10	
Brand	Xeomin	194	4.23	2.38	3.89	4.56	1	10	**0.027**
	Dysport	535	3.81	2.19	3.63	4.00	0	10	
Sex	Female	667	3.94	2.25	3.77	4.11	0	10	0.510
	Male	62	3.74	2.18	3.19	4.30	1	9	

*SD* standard deviation, *CI* confidence interval, *Min* minimum, *Max* maximum.

Significant values are in bold.

**Table 3:** Descriptive statistics and 95% CIs for pain intensities in each of the subgroups.

Dilution	Brand	Sex	N	Mean	SD	95% CI		Min	Max
Lidocaine	Xeomin	Female	56	3.36	1.76	2.89	3.83	1	9
		Male	5	3.80	3.03	0.03	7.57	1	8
		Both	61	3.39	1.86	2.92	3.87	1	9
	Dysport	Female	186	3.59	2.09	3.28	3.89	0	9
		Male	16	3.13	2.13	1.99	4.26	1	7
		Both	202	3.55	2.09	3.26	3.84	0	9
Saline (placebo)	Xeomin	Female	119	4.64	2.51	4.18	5.09	1	10
		Male	14	4.36	2.44	2.95	5.76	1	9
		Both	133	4.61	2.49	4.18	5.04	1	10
	Dysport	Female	306	3.99	2.26	3.73	4.24	1	10
		Male	27	3.78	1.93	3.02	4.54	1	7
		Both	333	3.97	2.24	3.73	4.21	1	10

*SD* standard deviation, *CI* confidence interval, *Min* minimum, *Max* maximum.

The Pearson coefficient showed a weak but statistically significant correlation between the individuals’ age and pain (R = 0.082, *P* = 0.026). The partial correlation coefficient (controlling for the dilution method, toxin brands, and participants’ sex) as well detected a weak but significant correlation between age and pain (R = 0.079, *P* = 0.032).

**Figure 4:**
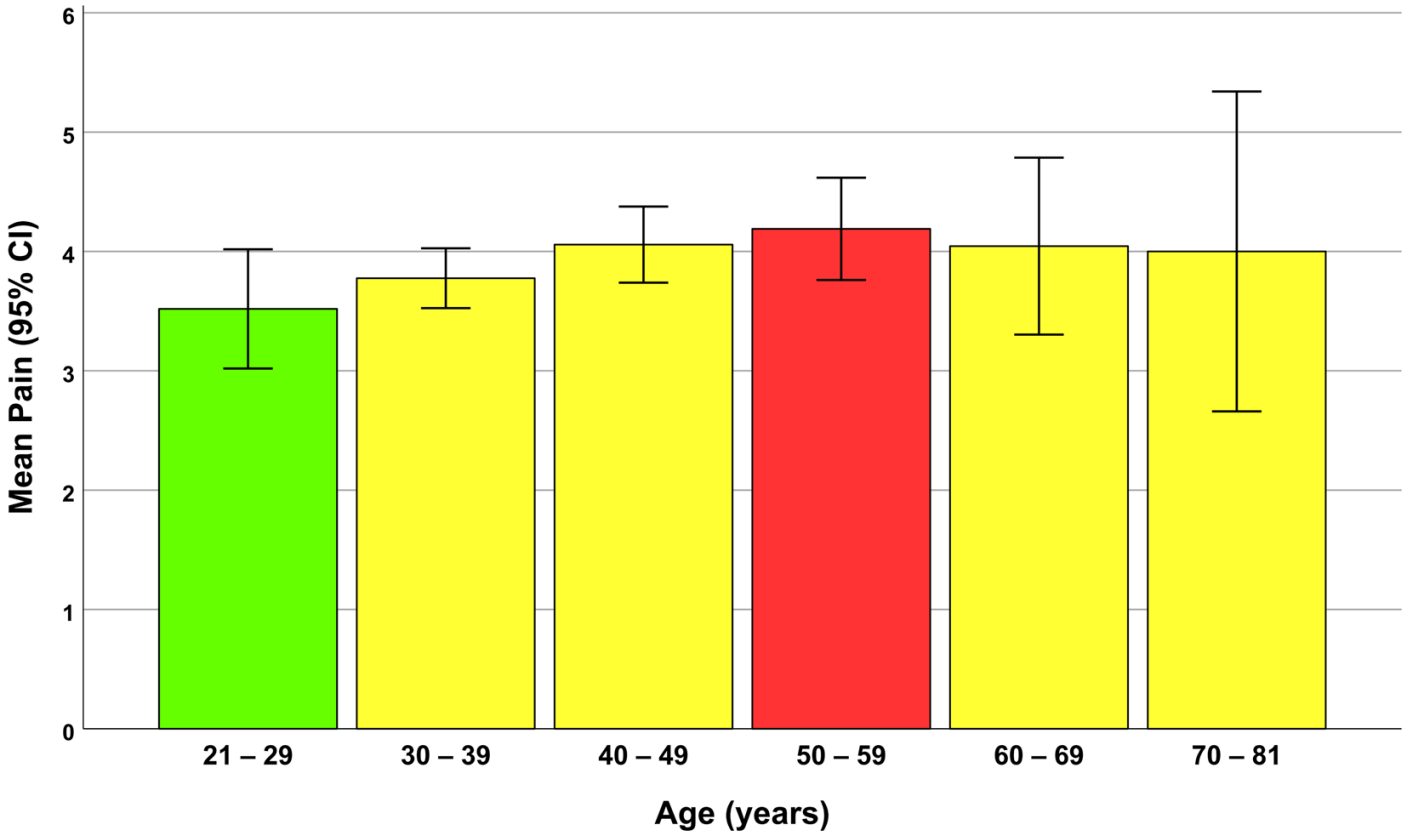
Means and 95% CIs for pain felt by participants in different decades of life. Note that these age groups are created merely for a more convenient understanding of how pain might change by age; they are not used for any statistical analyses. All statistical analyses of this study treated age as a continuous variable without any grouping. The green and red bars illustrate the life decades with the minimum and maximum average pain levels, respectively. Since there was only one participant in the life decade “80 to 89”, that individual (81 years old) was included in the age group of 70 to 81 years.

The distribution of patients’ ages is shown in Fig. 3. Moreover, descriptive statistics and 95% CIs for pain intensity felt by individuals at different decades of life as well as the incidence of their complications in different decades are presented in Fig. 4 and Table 4.

**Table 4:** Descriptive statistics and 95% CIs for pain levels felt by individuals at different decades of life, as well as the net incidence (and percentage) of 2-week complications in different decades of life.

Age	N	Pain						Incidence of complications (%)		
		Mean	SD	95% CI		Min	Max	Asymmetry	Retouch need	Ptosis
21–29	54	3.52	1.83	3.02	4.02	1.00	8.00	4 (7.41)	5 (9.26)	0
30–39	294	3.78	2.18	3.52	4.03	1.00	10.00	11 (3.74)	30 (10.20)	0
40–49	210	4.06	2.35	3.74	4.38	1.00	10.00	7 (3.33)	22 (10.48)	2 (0.95)
50–59	111	4.19	2.28	3.76	4.62	0.00	10.00	10 (9.01)	15 (13.51)	2 (1.80)
60–69	45	4.04	2.47	3.30	4.79	1.00	10.00	6 (13.33)	10 (22.22)	0
70–81	15	4.00	2.42	2.66	5.34	1.00	8.00	1 (6.67)	2 (13.33)	0

Since there was only one participant in the life decade “80 to 89”, that person (81 years old) was included in the age group of 70 to 81 years. Note that these age groups are only for a more convenient understanding of the pain levels and the percent of complications at different decades of age and not used for any statistical analyses. All statistical analyses treated age as a continuous variable without any grouping.

*SD* standard deviation, *CI* confidence interval, *Min* minimum, *Max* maximum.

**Figure 5:**
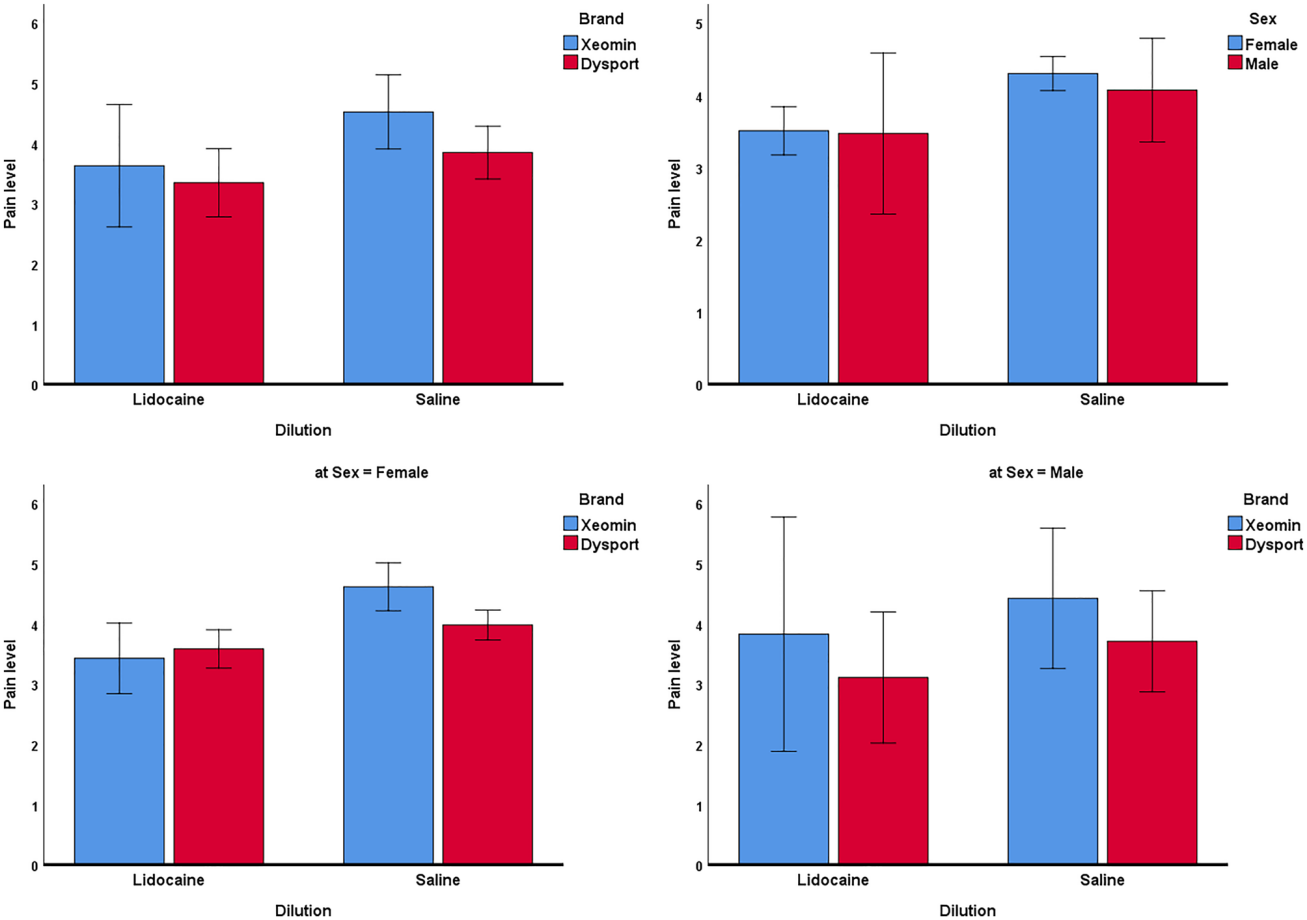
Estimated marginal means and 95% CIs for pain levels perceived in different subgroups. The marginal means are estimated at the average age of 42.2 years.

The full-factorial model of the 3-way ANCOVA identified age and BTX-A as factors influencing pain: age (*P* = 0.051), sex (*P* = 0.704), BTX-A brand (*P* = 0.177), dilution material (lidocaine versus placebo, *P* = 0.049), the interaction of sex by brand (*P* = 0.499), the interaction of sex by dilution material (*P* = 0.785), the interaction of brand by dilution material (*P* = 0.578), and the interaction of sex by brand by dilution material (*P* = 0.574). Since the interactions were all non-significant (*P* ≥ 0.499), they were removed from the model. The optimized ANCOVA model showed significant effects for the dilution material (lidocaine versus placebo [both botulinum brands combined], *P* = 0.001, Fig. 5), botulinum toxin brands (*P* = 0.030), and age (*P* = 0.032). Sex did not have a significant role (*P* = 0.406, Fig. 5).

### Complications

After 2 weeks, diplopia was not seen in any of the 729 participants.

#### Effects of the dilution materials

Complications were not significantly affected by placebo versus lidocaine. Ptosis was observed in 3 (0.6%) placebo participants and 1 (0.4%) lidocaine patient (*P* = 1.0, Fisher). Asymmetry was detected in 30 (6.4%) subjects within the placebo group and 9 (3.4%) cases within the lidocaine group (*P* = 0.089, Fisher). A botulinum toxin retouch was needed in 55 (11.8%) placebo cases and 29 (11.0%) lidocaine patients (*P* = 0.810, Fisher exact test, Table 5).

**Table 5:** Net frequency and percent of various 2-week complications observed in different subgroups.

Dilution	Brand	Sex	Ptosis (%)		Asymmetry (%)		Retouch (%)	
			No	Yes	No	Yes	No	Yes
Lidocaine	Xeomin	Female	56 (100)	–	55 (98.2)	1 (1.8)	55 (98.2)	1 (1.8)
		Male	5 (100)	–	5 (100)	–	5 (100)	–
	Dysport	Female	185 (99.5)	1 (0.5)	179 (96.2)	7 (3.8)	160 (86)	26 (14)
		Male	16 (100)	–	15 (93.8)	1 (6.3)	14 (87.5)	2 (12.5)
Saline (placebo)	Xeomin	Female	118 (99.2)	1 (0.8)	111 (93.3)	8 (6.7)	105 (88.2)	14 (11.8)
		Male	14 (100)	–	14 (100)	–	13 (92.9)	1 (7.1)
	Dysport	Female	304 (99.3)	2 (0.7)	284 (92.8)	22 (7.2)	269 (87.9)	37 (12.1)
		Male	27 (100)	–	27 (100)	–	24 (88.9)	3 (11.1)

#### Effects of the brand of botulinum toxin

Ptosis was observed in 1 (0.5%) Xeomin and 3 (0.6%) Dysport cases. Asymmetry was seen in 9 (4.6%) Xeomin and 30 (5.6%) Dysport participants. A BTX-A retouch was needed in 16 (8.2%) Xeomin and 68 (12.7%) Dysport patients. Brands had no significant effect on any of the complications (asymmetry: *P* = 0.712; a need for a retouch: *P* = 0.115; ptosis: *P* = 1.0; Fisher, Table 5).

#### Effects of sex

Four females (0.6%) and no males had ptosis. Asymmetry was observed in 38 females (5.7%) and 1 male (1.6%). A retouch was needed in 78 females (11.7%) and 6 males (9.7%). There was no difference between males and females in terms of any of the complications (asymmetry: *P* = 0.241; the need for a retouch: *P* = 0.835; ptosis: *P* = 1.0; Fisher, Table 5).

#### Effects of age

The point-biserial correlation coefficient showed that in the 729 patients, age (as a continuous variable without any grouping) was not correlated with ptosis (R = 0.042, *P* = 0.261). However, it was weakly correlated with asymmetry (R = 0.091, *P* = 0.014) and the need for a retouch (R = 0.080, *P* = 0.030).

#### Simultaneous effects of all variables

The multiple binary logistic regression did not show any significant effect of the dilution material, botulinum toxin brand, sex, and age (as a continuous variable without any grouping) on the incidence of ptosis (Table 6). However, in terms of asymmetry and a need for a retouch, it detected significant effects for age only (Table 6).

**Table 6:** The results of the multiple binary logistic regression analyses.

Complication	Predictor	B	SE	*P*	OR	95% CI for OR	
Ptosis	Sex	− 16.12	5004.66	0.997	0.000	0.000	
	Age	0.04	0.04	0.282	1.044	0.966	1.128
	Brand	0.09	1.17	0.940	1.091	0.111	10.713
	Dilution	0.44	1.17	0.708	1.551	0.156	15.415
	Constant	− 7.93	3.50	0.023			
Asymmetry	Sex	− 1.37	1.03	0.182	0.255	0.034	1.901
	Age	0.03	0.01	**0.022**	**1.032**	**1.005**	**1.060**
	Brand	0.21	0.39	0.589	1.237	0.571	2.678
	Dilution	0.62	0.39	0.112	1.865	0.865	4.024
	Constant	− 5.63	1.18	0.000			
Retouch	Sex	− 0.23	0.45	0.607	0.794	0.329	1.914
	Age	0.02	0.01	**0.039**	**1.021**	**1.001**	**1.042**
	Brand	0.46	0.29	0.117	1.584	0.892	2.814
	Dilution	0.07	0.25	0.784	1.070	0.660	1.733
	Constant	− 3.86	0.80	0.000			

***B*** regression coefficient, ***SE*** standard error, ***OR*** odds ratio, ***CI*** confidence interval. Age is a continuous variable without any grouping. The unit of change is 1 year for age; i.e. the age’s OR is computed for 1 year increase of age. The categorical variables were coded as follows: **Sex**: Female [0], Male [1]; Brand: Xeomin [1], Dysport [2]; Dilution: Lidocaine [1], Placebo [2].

Significant values are in bold.

## Discussion

The findings of this study indicated that the addition of lidocaine (without epinephrine) to botulinum toxin might reduce pain while at the same time, might not increase any complications of injection. There is no similar study that assesses the findings of the current study. There are however, two clinical series and two controversial clinical trial studies on the effect of dilution of botulinum toxin with lidocaine plus epinephrine^3,18,25,26^: a clinical trial of 10 patients only on complications but not pain, a clinical trial of 15 patients only on pain incidence, and two clinical series without any control groups. The clinical trials were as small as 10 and 15 participants^18,25^, and only one had examined pain^25^. One of them suggested that the incorporation of lidocaine and epinephrine does not make the injection outcomes more predictable, nor would it reduce the incidence of pain^25^; they did not assess the intensity of pain^25^. The other reported that the addition of lidocaine and epinephrine would make the toxin’s effect appear immediately and therefore increase predictability; they did not assess pain^18^. Since some of such effects do not appear immediately but after a delay (hours^27^ to even 2 weeks^3^), it may be difficult to accurately evaluate the results (such as any symmetries) at the time of injection, and therefore to provide symmetric results^3,16^. Hence, methods that can speed up the effects are of value. The longitudinal studies found lidocaine-epinephrine incorporation satisfactory^3,26^; however, no control existed to compare their methods. Ravso and Bove^26^ reported a series of cases with the injection of lidocaine with epinephrine plus botulinum toxin (without any control groups) and asserted that the incorporation of lidocaine might avoid imperfect results following the injection of botulinum toxin^26^. Kim et al.^3^ conducted another case series study and concluded that diluting botulinum toxin with lidocaine and epinephrine might improve patient satisfaction with injections for facial rejuvenation, due to faster results, longer effects, less bruising and pain, and improved cosmesis^3^. de Quadros et al.^25^ randomized the facial sides of 15 patients to receive botulinum toxin diluted with normal saline or reconstituted with lidocaine and epinephrine; in their study, 3 (20.0%) patients reported pain regarding the side treated with lidocaine, whereas 8 (53.3%) reported pain in the side incorporated with normal saline. Although this difference did not reach the significance level (*P* = 0.180), there might be a chance to see a statistically significant result if a larger number of patients had been enrolled. They also did not show any statistically significant differences in the frequency of muscle paralysis (although there seemed to be a notable difference in the first 48 h) and facial symmetry in the application of botulinum toxin diluted merely with saline solution compared to that diluted with lidocaine and epinephrine diluted^25^. Gassner and Sherris^18^ assessed the effects of the incorporation of lidocaine plus epinephrine into botulinum toxin on the predictability of botulinum injection in 10 patients^18^. They reported that the addition of lidocaine with epinephrine could provide the physician with immediate feedback on the extent of paralysis, and thus improve the predictability and safety of botulinum toxin^18^. As the closest study to our design in terms of the diluents of botulinum, Jung and Kim^28^ examined the intensity of pain after the injection of botulinum toxin diluted with lidocaine plus sodium bicarbonate in 20 patients and found pain-decreasing effects for the lidocaine-bicarbonate complex^28^.

Upon injection of botulinum toxin, pain can be felt in two stages, once when the needle punctures the skin and tissues and later after the toxin is injected into the tissue. The needle pain can be alleviated by locally anesthetizing the skin using methods such as the EMLA (eutectic mixture of local anesthetics) cream or ice packs^29^. The pain caused by the release of the toxin into the tissues can be reduced via anesthetics such as lidocaine. However, lidocaine incorporated into the toxin does not reduce the needle pain. Therefore, in clinical settings, it is recommended to reduce pain in the early and late phases. It seems that pain occurs mainly on the skin and therefore can be related to the needle gauge and the technique of injection. This could be a reason that when the same person with the same technique injects botulinum toxin, the pain might be similar. However, this hypothesis needs future investigations. Another point is that it is suggested that lidocaine might cause burning sensations and therefore increase pain^30^. This can in turn be responsible for the observed results, as a confounder. On the other hand, it is said that lidocaine might increase the speed of botulinum action because it can increase blood flow and also numb the muscle and allow it to absorb more botulinum. This as well warrants future research.

This study was limited by some factors. The number of men was much smaller than the number of women; this was similar to all other studies on botulinum toxin, in which much more women seek such treatments. However, given the very large sample size, even the current number of men may be adequate to obtain proper results. Moreover, the number of one of the brands in use was smaller than the other brand, due to market availability. Furthermore, it would be better to also have positive control groups, in which the toxin was mixed with lidocaine and adrenaline. Nevertheless, that would require about 200 to 300 additional participants, which was not practical. The generalizability of the results may be increased by using various brands of botulinum toxin and enrolling more men. Nevertheless, it may be limited by the ethnic background of the participants, as in any other study.

## Conclusions

It may be concluded, for the first time, that the addition of lidocaine without epinephrine or any other chemicals to the BTX-A may reduce the pain perceived by the individual during botulinum toxin administration. However, it does not affect the postinjection complications observed after 2 weeks. The injection of the Xeomin brand would be more painful than Dysport, but it might not affect the 2-week complications. People’s age might slightly increase the injection pain as well as two of the complications, i.e. asymmetry and the need for a botulinum toxin retouch. Their sex might not affect pain levels or 2-week post-injection complications.

## Data Availability

The raw data are available from the corresponding author upon request.
